# Dentate gyrus granule cell activation following extracellular electrical stimulation: a multi-scale computational model to guide hippocampal neurostimulation strategies

**DOI:** 10.3389/fncom.2025.1638002

**Published:** 2025-08-01

**Authors:** Shayan Farzad, Tianyuan Wei, Jean-Marie C. Bouteiller, Gianluca Lazzi

**Affiliations:** ^1^Department of Biomedical Engineering, University of Southern California, Los Angeles, CA, United States; ^2^Institute for Technology and Medical Systems (ITEMS), Keck School of Medicine, University of Southern California, Los Angeles, CA, United States; ^3^Department of Electrical Engineering, University of Southern California, Los Angeles, CA, United States; ^4^Department of Ophthalmology, Keck School of Medicine, University of Southern California, Los Angeles, CA, United States

**Keywords:** extracellular electrical stimulation, admittance method, electrode design, multiscale modeling, neuron

## Abstract

**Introduction:**

The effectiveness of neural interfacing devices depends on the anatomical and physiological properties of the target region. Multielectrode arrays, used for neural recording and stimulation, are influenced by electrode placement and stimulation parameters, which critically impact tissue response. This study presents a multiscale computational model that predicts responses of neurons in the hippocampus—a key brain structure primarily involved in memory formation, especially the conversion of short-term memories into long-term storage—to extracellular electrical stimulation, providing insights into the effects of electrode positioning and stimulation strategies on neuronal response.

**Methods:**

We modeled the rat hippocampus with highly detailed axonal projections, integrating the Admittance Method to model propagation of the electric field in the tissue with the NEURON simulation platform. The resulting model simulates electric fields generated by virtual electrodes in the perforant path of entorhinal cortical (EC) axons projecting to the dentate gyrus (DG) and predicts DG granule cell activation via synaptic inputs.

**Results:**

We determined stimulation amplitude thresholds required for granule cell activation at different electrode placements along the perforant path. Membrane potential changes during synaptic activation were validated against experimental recordings. Additionally, we assessed the effects of bipolar electrode placements and stimulation amplitudes on direct and indirect activation.

**Conclusion:**

Stimulation amplitudes above 750 μA consistently activate DG granule cells. Lower stimulation amplitudes are required for axonal activation and downstream synaptic transmission when electrodes are placed in the molecular layer, infra-pyramidal region, and DG crest.

**Significance:**

The study and underlying methodology provide useful insights to guide the stimulation protocol required to activate DG granule cells following the stimulation of EC axons; the complete realistic 3D model presented constitutes an invaluable tool to strengthen our understanding of hippocampal response to electrical stimulation and guide the development and placement of prospective stimulation devices and strategies.

## 1 Introduction

Interfacing with neural systems often employs invasive electrode-based methods for stimulation and recording. The development of implantable medical devices using multi-electrode arrays (MEAs) marks a significant achievement in neural engineering. MEAs enable the recording and stimulation of a large number of neurons with increased spatial and temporal precision (Zhou et al., 2015; Hsiao et al., 2015; Heuvelmans et al., 2024; Spira and Hai, 2013), enabling more refined modulation of neuronal activity and paving the road toward the development of hippocampal memory prostheses (Hsiao et al., 2015). Recent advancements in medical implants and MEAs in particular are trending toward miniaturization and increased electrode density (Spira and Hai, 2013; Cogan, 2008; Brunner et al., 2011). Yet, maximizing the benefits of these technological advances would strongly benefit from a deep understanding of the targeted neural system's electrophysiology and anatomy, the topology of the neural networks targeted, and the interactions between the exogenous electric field applied and the neural tissue. Taking into consideration this comprehensive knowledge constitutes a great resource toward optimizing MEA design, determining optimal electrodes placement, orientation, electrode number and density, and stimulation patterns to elicit the desired neural responses. The hippocampus plays a crucial role in learning and memory formation. Damage to the hippocampal region is known to result in memory impairment, dramatically affecting quality of life (Mitchell et al., 2010). Ongoing research is focused on developing a hippocampal prosthesis aimed at functionally replacing parts of the hippocampal neural circuitry that no longer function appropriately, with substantial progress made in predicting and replicating the spatiotemporal patterns of activity as they propagate in the hippocampus (Berger et al., 2011). Additionally, recent research indicates that patterns of electrical stimulation may also have therapeutic potential for treating cognitive disorders (Wu et al., 2024).

Given the intricate anatomy of the hippocampus, a priori investigation of the consequences of a specific MEA design, and more specifically the placement of the electrodes with respect to the neurons to be activated may help identify the optimal range for stimulation parameters and overall strategy to use to elicit the desired neural response. Recent advances in computational methodologies, along with increased computational power have led to the development of highly predictive models which have successfully informed the design of several prosthetic devices and examined the impacts of exogenous electrical stimulation (Loizos et al., 2016a; Farzad et al., 2023; Paknahad et al., 2021; Loizos et al., 2016b; Howell et al., 2014; Howell and Grill, 2014; Tsai et al., 2012; Howell et al., 2015; Martens et al., 2011; Mcintyre and Grill, 1999; Miocinovic et al., 2009; Foutz and McIntyre, 2010) and evaluated the precision of modern methodologies and parameters (Grill, 1999; Howell and McIntyre, 2017; Butson and McIntyre, 2005). Although these models have provided valuable insights, achieving sufficient biological realism specific to the hippocampal region is essential for accurately evaluating the electrode arrays' ability to induce the spatiotemporal patterns of neuronal activation desired in this intricate neural system. To address this, we have developed a multiscale model that integrates a highly realistic large-scale neuronal network of the rat hippocampus (Yu et al., 2014) with stimulating and/or recording electrodes inserted in the tissue using an extensive, three-dimensional Admittance Method-NEURON model (Loizos et al., 2016a; Farzad et al., 2023; Paknahad et al., 2021; Loizos et al., 2016b; Hines and Carnevale, 1997; Cela, 2010; Bingham et al., 2018; Eberdt et al., 2003).

At the tissue level, accurately representing the extracellular space is crucial to accurately predict the spatial distribution of current within tissue in a stimulating or recording system. Hence, accurate dielectric properties are essential for discretizing the space into circuit components and creating distinct anatomical representations of the tissue (Grill, 1999; Howell and McIntyre, 2017). Grill and McIntyre have shown that Rattay-style activating functions fall short in accurately predicting action potential initiation sites, thus highlighting the significant advantages of using detailed neural models for neurons (Mcintyre and Grill, 1999). Numerous computational models of neurons have been developed for the brain structures of interest, each with varying degrees of realism. Yet, it remains unclear whether simpler models than those described in this paper can predict neural circuit responses to extracellular electrical stimulation with the desired level of accuracy (Herz et al., 2006). Given that different neural components (dendrites, somas, and axons) are differentially activated, and that axonal chronaxie is significantly shorter than somatic chronaxie (by a factor of up to 40), using detailed and realistic axonal morphologies appears of paramount importance. This approach should yield better predictions of the spatial and anatomical specificity of the neural activity in response to electrical stimulation (Mcintyre and Grill, 1999; Nowak and Bullier, 1998).

The hippocampus is a highly organized structure with distinct anatomical pathways, including the trisynaptic circuit (EC → DG → CA3 → CA1), which supports episodic memory encoding, spatial navigation, and context discrimination (Andersen et al., 1971; Amaral and Witter, 1989). The entorhinal cortex, which provides the major cortical input to the hippocampus, can be functionally divided into the medial (MEC) and lateral (LEC) subdivisions. These regions contribute differently to hippocampal processing: MEC neurons convey spatial and path integration signals (e.g., grid cells, head-direction cells), while LEC neurons primarily encode object identity and non-spatial sensory information (Deshmukh and Knierim, 2011; Knierim et al., 2014; Hargreaves et al., 2005). Their axons project to distinct laminar zones within the molecular layer of the dentate gyrus, enabling region-specific modulation of granule cell activity. Despite extensive computational modeling of CA1 and CA3 pyramidal cells, relatively few studies focus on the dentate gyrus and its input layer under the influence of localized stimulation. Prior models of this region have largely depended on stochastic representations of entorhinal input activity, such as probabilistic firing of axons, to estimate granule cell responses across different laminar domains (Aimone et al., 2011; Santhakumar et al., 2005). However, these approaches do not capture the spatially heterogeneous distribution of extracellular electric fields that result from targeted electrical stimulation. To address these limitations, our model integrates a circuit-based volume conductor approach with high spatial resolution, enabling more realistic simulations of electrical field spread and its localized impact on dentate circuitry.

Entorhinal cortical inputs provide the primary excitatory drive to the dentate gyrus, conveying sensory and contextual information from the neocortex (Witter et al., 2000). Granule cells, in turn, transform these inputs through sparse firing and high activation thresholds, enabling effective pattern separation (Jung and McNaughton, 1993; Rolls, 2010). This selective activation constitutes an opportunity for examining the initial stages of hippocampal signal flow and allows for localized control over stimulation, making it particularly useful for studying the effects of spatially targeted neuromodulation (Aimone et al., 2011). Our model constitutes an ideal framework to perform these investigations as it integrates both exogenous electrical stimulation and morphologically realistic neural network.

Within this context, this study systematically explores the activation of realistic entorhinal cortical (EC) axons in the perforant path resulting from electrical stimulation by electrodes placed in the perforant path, which, in turns, indirectly activate granule cells in the dentate gyrus. By simulating different electrode locations and stimulus amplitudes, we investigate how both anatomy and stimulus strength individually and collectively influence granule cell responses.

## 2 Methods

The neural response to extracellular electric fields is modeled using a computationally realistic representation of a rat dentate gyrus (DG). This specific model focuses on over 8,000 entorhinal cortical (EC) axons that project onto 11 adjacent granule cells (GC) (located, on average, within 75 μm of each other). The various methodologies and parameters used in the development of this model are described in prior studies (Hendrickson et al., 2015; Tamamaki and Nojyo, 1993). The major simulation stages are the following: (1) A 3D model of hippocampal tissue is used to calculate the electric field distribution resulting from bipolar electrode stimulation; (2) this field is then applied to the different segments of the neuron models, which activation is computed to determine the neural response to the electrical stimulation.

### 2.1 Computational tools and infrastructure

The admittance method (Loizos et al., 2016a; Farzad et al., 2023; Paknahad et al., 2021; Loizos et al., 2016b; Bingham et al., 2018; Eberdt et al., 2003) determines the field distribution, while neural network simulations are performed using compartmental models within the NEURON simulation environment (version 7.7). The model runs in parallel on a computing cluster with 4,040 processors, supported by the High-Performance Computing and Communications Center at the University of Southern California. The analysis of the axonal projections to a single GC required 40 nodes corresponding to 2,000 CPUs and 1 GB of RAM per CPU.

### 2.2 Establishing anatomical reference coordinates

In 2018, Bingham et al. (2018) developed a computational model that simulates the effects of electrical stimulation *in-vitro* on a 400 μm thick hippocampal slice. The current model builds upon this and extends the 2D extruded slide model to include the entire septotemporal extent of the dentate gyrus, with granule cell (GC) neurons and their corresponding dendritic morphologies.

To do this, we use a detailed 3D hippocampal model obtained by serial tracing of high-resolution thin histological sections of a rat brain, combined with dense digital embedding of reconstructed neuronal morphologies (Ropireddy et al., 2012). This 3D model allows us to quantify the volumetric distributions and dendritic occupancy within the hippocampal layers and subregions, which are mapped to standard brain coordinates and hippocampal axes. Although other well-established rodent brain atlases, like those by Swanson, Paxinos or Kjonigsen, are available, we chose to use the Ropireddy atlas for this work because it offers marginally higher resolution and granularity throughout the entire hippocampal extent and provides more detailed region demarcation within the hippocampus (Kjonigsen et al., 2011; Petrovich et al., 2001; Vogt and Paxinos, 2014).

**Figure 1:**
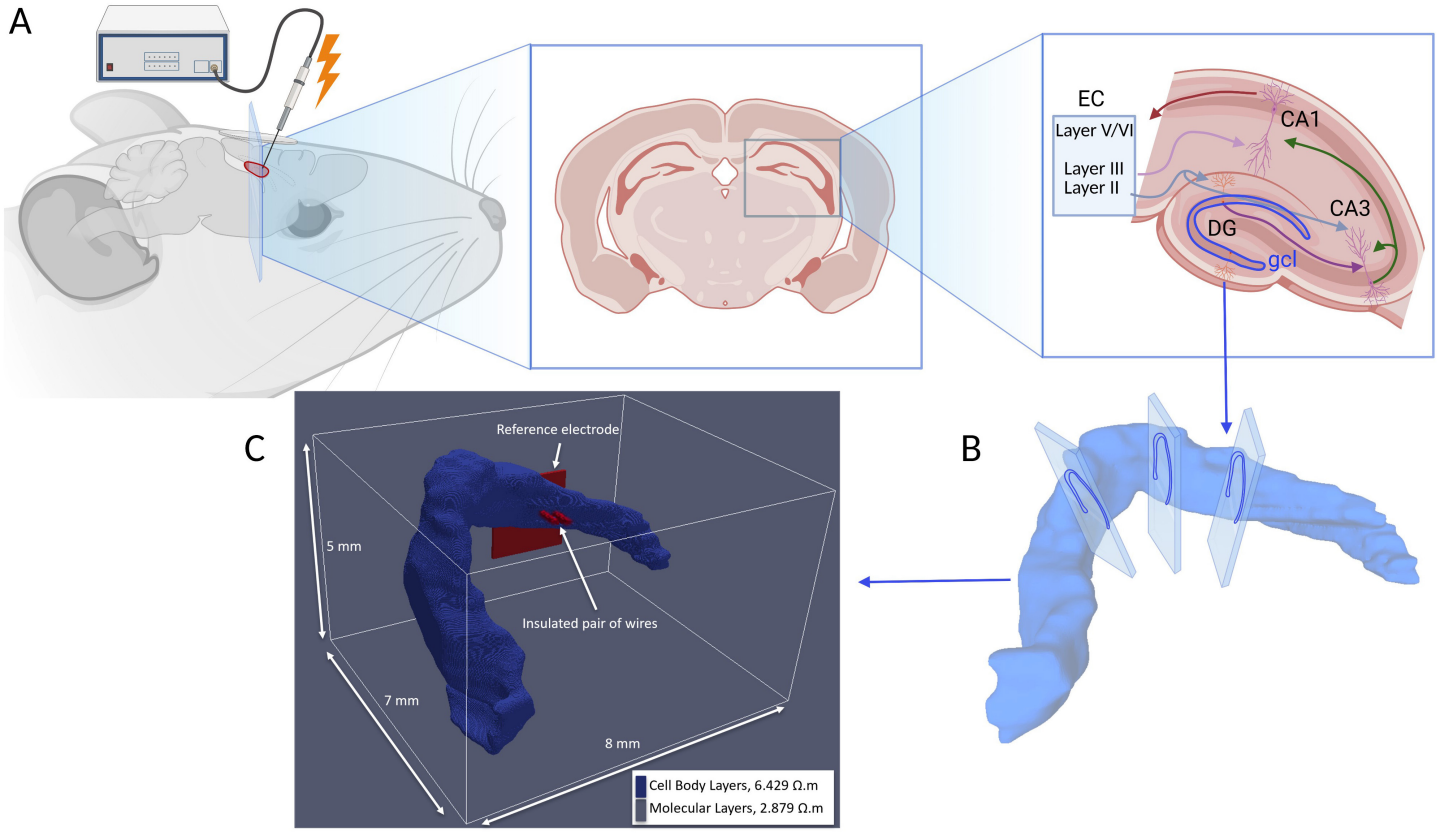
**(A)** Illustration depicting electrical stimulation of the hippocampal region with a diagram of the hippocampo-entorhinal circuit, illustrating the main trisynaptic pathway EC → DG → CA3 → CA1. CA, cornu ammonis; DG, dentate gyrus; EC, entorhinal cortex. **(B)** Serial tracing of high-resolution thin histological sections of a rat brain (Ropireddy et al., 2012) has been converted into **(C)** the three-dimensional voxel grid which is utilized as the tissue model in the AM-NEURON multiscale simulation, enabling detailed computational modeling of neuronal activity within this anatomically accurate hippocampal tissue model. Experimentally obtained resistivity values are applied to cell body regions (blue) and molecular layers (remaining of the model space, not shown) of DG (López-Aguado et al., 2001).

Furthermore, we employ this detailed 3D hippocampal model as the foundational tissue volume for constructing the AM-NEURON multiscale simulation. To integrate the anatomical data into the simulation environment, we convert these regions into a voxelized format. By accurately assigning to each voxel the corresponding resistivity value, we create a precise 3D representation of the hippocampal tissue, illustrated in Figure 1. The resulting three-dimensional voxel grid is then utilized as the tissue model in the AM-NEURON multiscale simulation, enabling detailed computational modeling of neuronal activity within this anatomically accurate hippocampal tissue model.

### 2.3 Admittance method model construction and NEURON interface (AM-NEURON)

The Admittance Method (AM) was selected over other techniques like the Finite Element Method (FEM) because it offered specific advantages pertinent to this study. In particular, this method is simpler to implement than FEM. Although FEM supports non-uniform grids, its computational complexity increases with more independent current sources. In contrast, an AM model may efficiently include multiple current sources, making it practical for modeling multi-electrode arrays and estimating feedback potentials. Second, representing the model space as an electrical circuit offers an intuitive way to model bulk neural tissue. This approach aligns naturally with neuron models, which also use circuit elements, enabling voltages within the volume conductor to serve as external inputs to neurons. As a result, both the tissue and neural networks are described within a unified circuit framework.

To calculate the extracellular voltage generated by electrical stimulation, we build a model of hippocampal tissue along with a bipolar stimulating electrode. A highest resolution of 10 μm is chosen to provide a reasonable amount of spatial detail for accurately capturing axonal compartments. A coarser resolution (up to 640 μm) is used in bulk tissue surrounding the model to reduce computational load. Each voxel is assigned specific material properties, as described in the following section. As current is injected into the virtual electrode, we compute the voltage at each voxel node within the 3D tissue model using AM. For further details pertaining to the AM method, we refer the reader to Loizos et al. (2016a), Farzad et al. (2023), Paknahad et al. (2021), Loizos et al. (2016b), Bingham et al. (2018), and Eberdt et al. (2003). The multicompartmental neuron morphologies that reside within the 3D tissue model receive the computed voltages as voltage applied extracellularly on their membrane. More specifically, we compute the extracellular voltage at each neuronal compartment as a linear interpolation of the voltage values at the nodes that define the voxel that contains a specific compartment and apply the result to that specific compartment. These calculations are done for all compartments of all neurons in the network.

**Figure 2:**
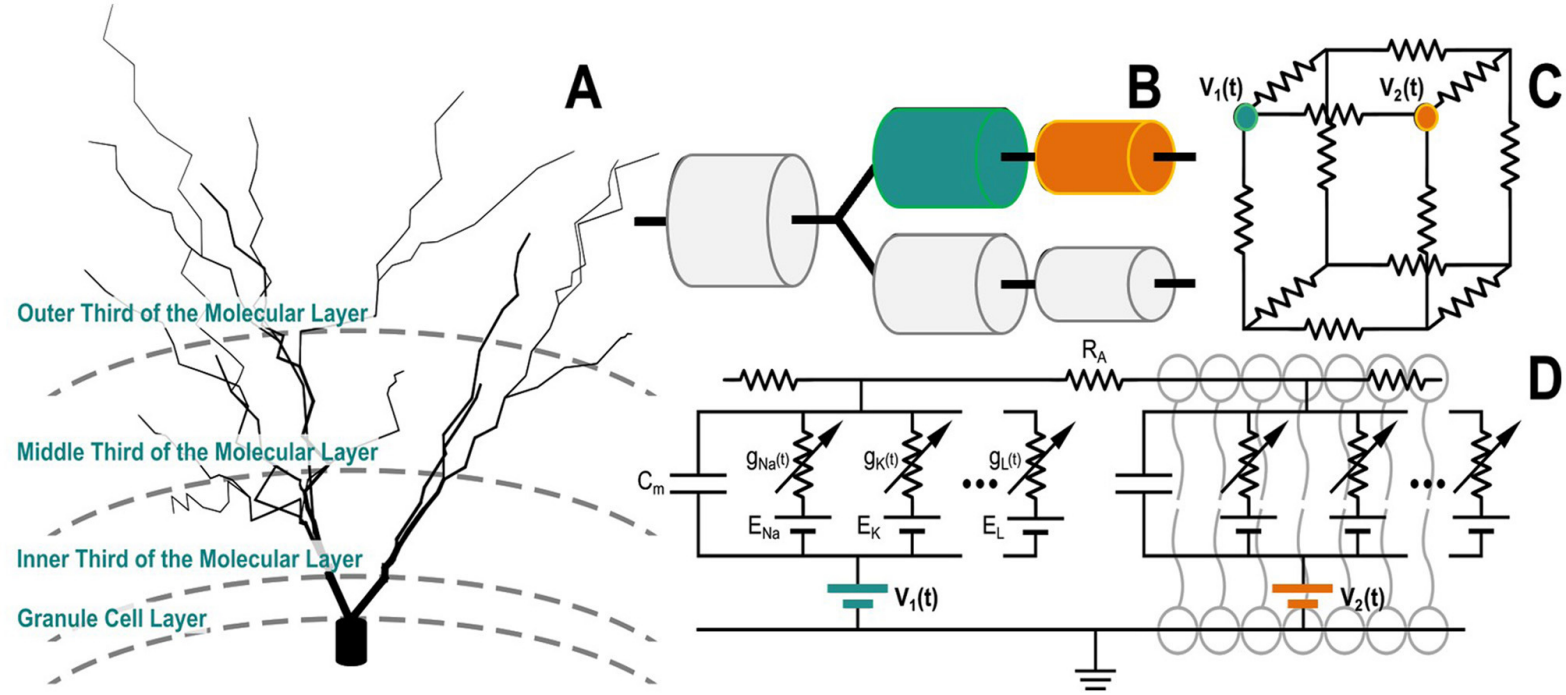
A granule cell compartmental model is depicted as a cable model, with compartments linked in series or parallel based on their morphology **(A, B)**. Each compartment is modeled as a circuit. **(C)** This diagram illustrates how the neuron compartmental model is integrated in the tissue simulated with the Admittance Method. Notably, this diagram shows the simple case where the two (teal and orange) compartments perfectly align with nodes of the voxeled AM model. In reality, compartments may reside at arbitrary positions within the computational volume. In such cases, the voltages from surrounding nodes are used to interpolate the extracellular potential to be applied at the exact position of each compartment; **(D)** illustrates the circuit equivalent representation of a two-compartment neuron model.

Given that the AM and NEURON models occupy the same coordinate space, the voltages from the AM are applied to the NEURON model to drive the neuronal compartments, as shown in Figure 2. By adding voltage sources in series with each membrane, extracellular potentials can be introduced to the neuronal compartments in the NEURON model, as described by the following relationship (Equation 1):


(1) $$\begin{array}{l} I_{m}\left(t\right)=\frac{dV_{ext}}{dt}\times C_{m} \end{array}$$

Where *I*_*m*_ is the transmembrane current resulting from an extracellular potential (*V*_*ext*_) that charges the membrane capacitance (*C*_*m*_).

### 2.4 Hippocampal tissue resistivity

The voxel model was discretized based on the resistive characteristics of hippocampal tissue at low frequencies (under 10 kHz). In the absence of impedance data for the dentate gyrus, resistivity values were assigned using measurements from the CA1 region. These measurements were conducted using the four-electrode impedance method by Lopez-Aguado, Ibarz, and Herreras (López-Aguado et al., 2001). These measurements revealed resistivity values of ~2.9 Ω·m in the molecular regions and 6.4 Ω·m in the cell body regions. Consequently, the computational model was simplified by dividing the hippocampus into two distinct regions: one representing the molecular area and the other the cell body area, each with uniform resistive properties based on these findings. The resistivity values utilized in the model correspond to those documented by Lopez-Aguado et al. and are illustrated in Figure 1.

Electrical stimulation was modeled by incorporating a pair of insulated microwire electrodes along with a reference electrode, following the experimental setup described by Soussou et al. (2006), as depicted in Figure 1. The electrodes, each with a diameter of 50 μm, had the resistivity of platinum (10^−8^ Ω·m).

The electrodes were encased in insulation that was 20 μm thick, possessing the dielectric properties of Teflon (10^16^ Ω·m).

A bipolar current source was used, with the anode in one microwire and the cathode in the other. Simulations were performed using a biphasic, square-wave pulse with a 1 ms duration. The results were then interpolated using tri-linear methods and applied to each segment of each neuron in the large-scale compartmental (NEURON) model. This process charges the membrane, generating intracellular currents, and the response of each cell in the network is then computed.

### 2.5 Granule cells (GC) and synapses

The GC models were created using the L-NEURON tool (Ascoli and Krichmar, 2000), with structural parameters derived from a database of GC morphological reconstructions to generate unique structures (Williams and Matthysse, 1983). Uniform compartment lengths were maintained, and the current models include the dendrites and somas, excluding the axons.

Biophysical parameters for the GCs were sourced from previously established computational models of the dentate gyrus (Santhakumar et al., 2005; Yuen and Durand, 1991). These parameters detail the ion channel types, densities, and distributions within the neuron model. The electrophysiological characteristics and parameters of the GC models have been validated in earlier research, as presented in Table 1 (Hendrickson et al., 2015).

**Table 1:** Passive and active properties for dentate granule cells.

**Passive property**	**Value**	**Mechanism**	**Soma**	**GCL**	**Inner 1/3**	**Middle 1/3**	**Outer 1/3**
R.M.P. (mV)	-78.02	Cm (μF/cm^2^)	9.8	9.8	15.68	15.68	15.68
R^*in*^ (MΩ)	242.2	Ra (ohm-cm^2^)	210	210	210	210	210
Membrane time constant (ms)	34.5	Leak (S/cm^2^)	2.9E-4	2.9E-4	4.6E-4	4.6E-4	4.6E-4
		Sodium (S/cm^2^)	0.84	0.126	0.091	0.056	-
		Delayed Rectifier K (slow) (S/cm^2^)	6.0E-3	6.0E-3	6.0E-3	6.0E-3	8.0E-3
		Delayed Rectifier K (fast) (S/cm^2^)	0.036	9.0E-3	9.0E-3	2.25E-3	2.25E-3
		A-type K (S/cm^2^)	0.108	-	-	-	-
		L-type Ca (S/cm^2^)	2.5E-3	3.8E-3	3.8E-3	2.5	-
		N-type Ca (S/cm^2^)	1.5E-3	7.4E-4	7.4E-4	7.4E-4	7.4E-4
		T-type Ca (S/cm^2^)	7.4E-5	1.5E-4	5.0E-4	1.0E-3	2.0E-3
		Ca-dependent K (S/cm^2^)	1.0E-3	4.0E-4	2.0E-4	-	-
		Ca- and V-dependent K (S/cm^2^)	1.2E-4	1.2E-4	2.0E-4	4.8E-4	4.8E-4
		Tau for decay on intracell. Ca (ms)	10.0	10.0	10.0	10.0	10.0
		Steady-state intracell. Ca (mol)	5.0E-6	5.0E-6	5.0E-6	5.0E-6	5.0E-6

EC axons connect to the GCs through 1900–2500 synapses (Eberdt et al., 2003) evenly located in the outer and middle thirds of the molecular layer, corresponding to the lateral perforant path and medial perforant path axons, respectively. When an action potential is triggered, it propagates down the axon and reaches the presynaptic terminal, leading to the release of excitatory neurotransmitters (in this case glutamate). The neurotransmitters then bind to ionotropic receptors triggering the opening of their associated channels, leading to a temporary increase in synaptic conductance and a depolarizing flow of ions. We model the conductance time course (*g*) using the difference between two exponential functions (Equation 2) (Tang et al., 2023):


(2) $$\begin{array}{l} g=e^{\frac{-t}{\tau_{1}}}-e^{\frac{-t}{\tau_{2}}} \end{array}$$

Notably, in this model, the postsynaptic response between entorhinal axons and GCs is mediated solely by AMPA receptors. The τ_1, 2_ are 1.05 and 5.75 ms for both medial (MEC) and lateral entorhinal cortical (LEC) axons with synaptic weights of 1.17E-4 and 1.5E-4 μS, respectively (Hendrickson et al., 2015).

### 2.6 Axonal projections of the perforant path from the entorhinal cortex

#### 2.6.1 3D reconstruction of axons

The model includes detailed 3D reconstructions of the rat dentate gyrus based on thin histological sections (Ropireddy et al., 2012). The entorhinal cortex projects to the hippocampus through the perforant pathway, with specific topographical distinctions between medial and lateral subdivisions. Upon reaching the dentate gyrus, the lateral perforant path terminates in the outer third of the molecular layer, while the medial perforant path terminates in the middle third (Hjorth-Simonsen and Jeune, 1972; Witter, 2007). The foundational study by Dolorfo and Amaral (Dolorfo and Amaral, 1998) served as a key reference in modeling the entorhinal cortex's projections to the dentate gyrus. Using retrograde dye injections into the dentate gyrus of rats, the study identified specific regions within the entorhinal cortex that send projections to those targeted areas in the dentate gyrus, thus clarifying the detailed topographical organization of these entorhinal connections.

Using the connectivity map of granule cells (GC) with perforant path axons, we identified the corresponding axon IDs from the medial and lateral entorhinal cortex (MEC and LEC) that connect to the target GC.

Ultimately, it also integrates the dentate gyrus GCs and axon arbors from the entorhinal cortex (EC), which connect to the GCs via the perforant path, with realistic axon arbor configurations generated using the Ruled-Optimum Ordered Tree System (ROOTS) algorithm (Bingham et al., 2020).

**Figure 3:**
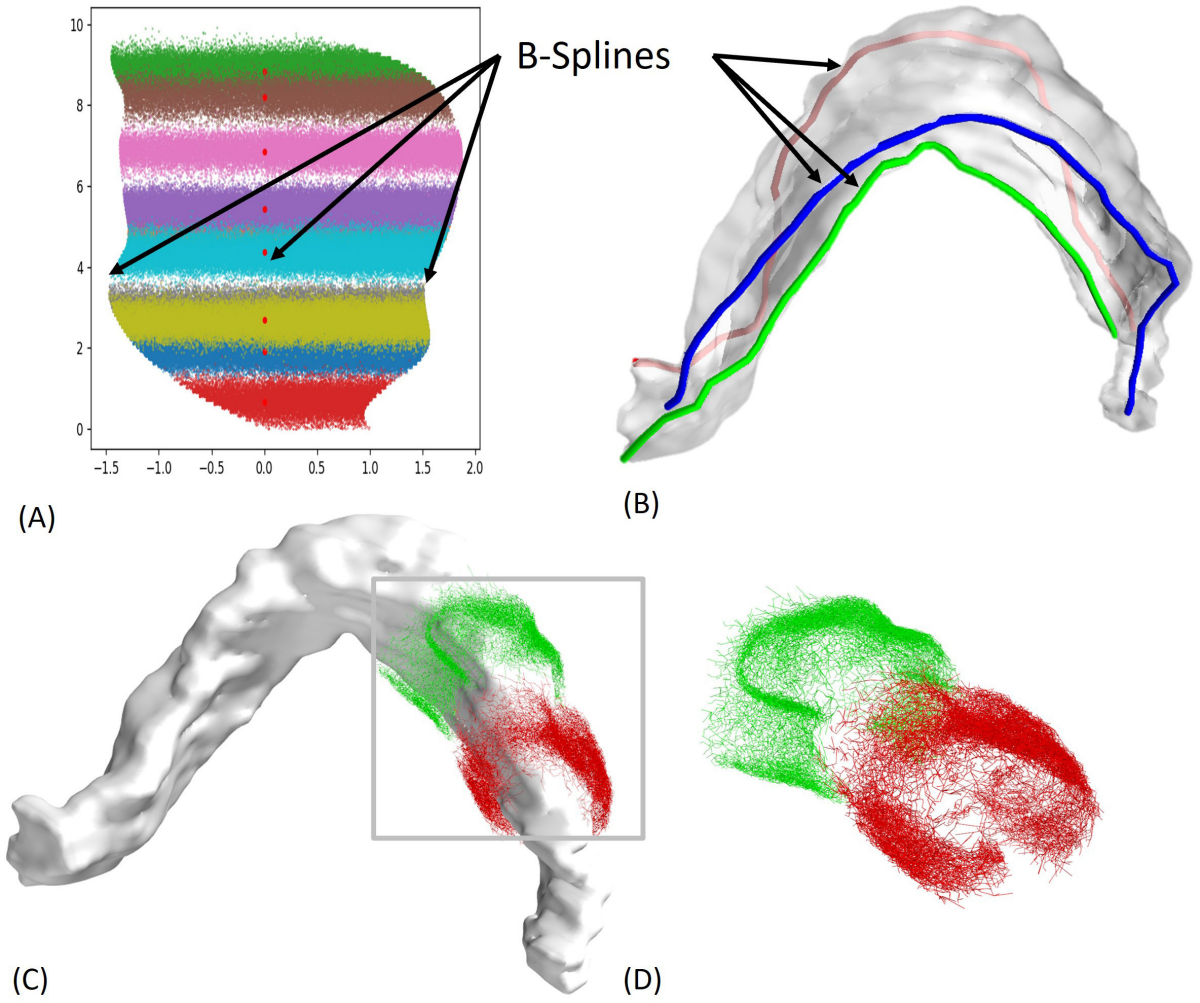
2D to 3D Mapping steps and axon arbor reconstruction. **(A)** The 2D locations of the granule cells for all entorhinal cortex (EC) axons were obtained from experimental work by (Gaarskjaer, 1978), with each axon having ~2,500 connections, **(B)** B-splines were used to map the 2D data onto a 3D model, **(C)** Reconstruction Using ROOTS Algorithm: Reconstructed axon arbors using the ROOTS Algorithm, ensuring accurate representation of axonal paths (Bingham et al., 2020), **(D)** Synaptic Connections: Over 1,900 axons make synaptic connections to a single granule cell. The two depicted axons illustrate the range of dentate gyrus (DG) coverage, representing the first and last EC axons connecting to the same granule cell.

The benefit of using ROOTS is that it enhances neuronal morphology generative techniques by incorporating intricately branched cortical axon terminals. It also excels at accurately reflecting biological realism within model fibers, improving the precision of predictions related to how microscale structures and branching configurations affect spatiotemporal activity patterns under extracellular electric fields. Figure 3 illustrates the 3D reconstruction steps and the complexity of the first and last EC axons that connect to a single granule cell. Of importance, there are over 1,900 more axons in between the two presented EC axons.

#### 2.6.2 Myelination

In the study by Bingham et al. (2018), the EC axons are modeled in a simplified manner, without myelination, and with a uniform diameter. However, recent studies indicate that hippocampal axons are, in fact, myelinated, which plays a critical role in their function (Meier et al., 2004; Harich et al., 2008; Aberra et al., 2018; Nickel and Gu, 2018).

Aberra et al. (2018) adapted neuron models from the Blue Brain Project (Markram et al., 2015; Ramaswamy et al., 2015) to better mirror the geometric and biophysical traits of adult rat and human cortical neurons. These adjustments were made to more accurately simulate their response to different types of stimulation. The model in this study, incorporated more accurate features of EC axons by including biophysical properties of myelinated L2/3 pyramidal cells. The EC axon was divided into segments along its length, with each segment consisting of a 20 μm myelinated portion followed by a 1 μm node of Ranvier.

Neuronal morphology plays a critical role in shaping responses to extracellular electrical stimulation. For example, axon diameter affects the input resistance and threshold for action potential initiation, thinner axons generally require higher field strength to activate (McIntyre et al., 2002). Axons in the cortical region, including those of the hippocampus, have been shown to taper as they bifurcate and extend toward their target region (Hu et al., 2009). Key morphological features, such as axon diameter, tapering, branching (bifurcations), and orientation relative to the electric field, significantly affect the degree of polarization and likelihood of activation. These features influence the spatial distribution of transmembrane potentials induced by the external field and, consequently, the excitability of different compartments, highlighting the importance of incorporating realistic morphologies in stimulation models. Based on this understanding, the diameter of the EC axons is assumed to taper from 1.6 μm at the origin to 0.8 μm at the final bifurcation along the axon. This tapering reflects more accurately what is observed in the biological structure, thereby more faithfully replicating the resulting functional properties of these EC axons as they connect with their target granule cells.

## 3 Results

**Figure 4:**
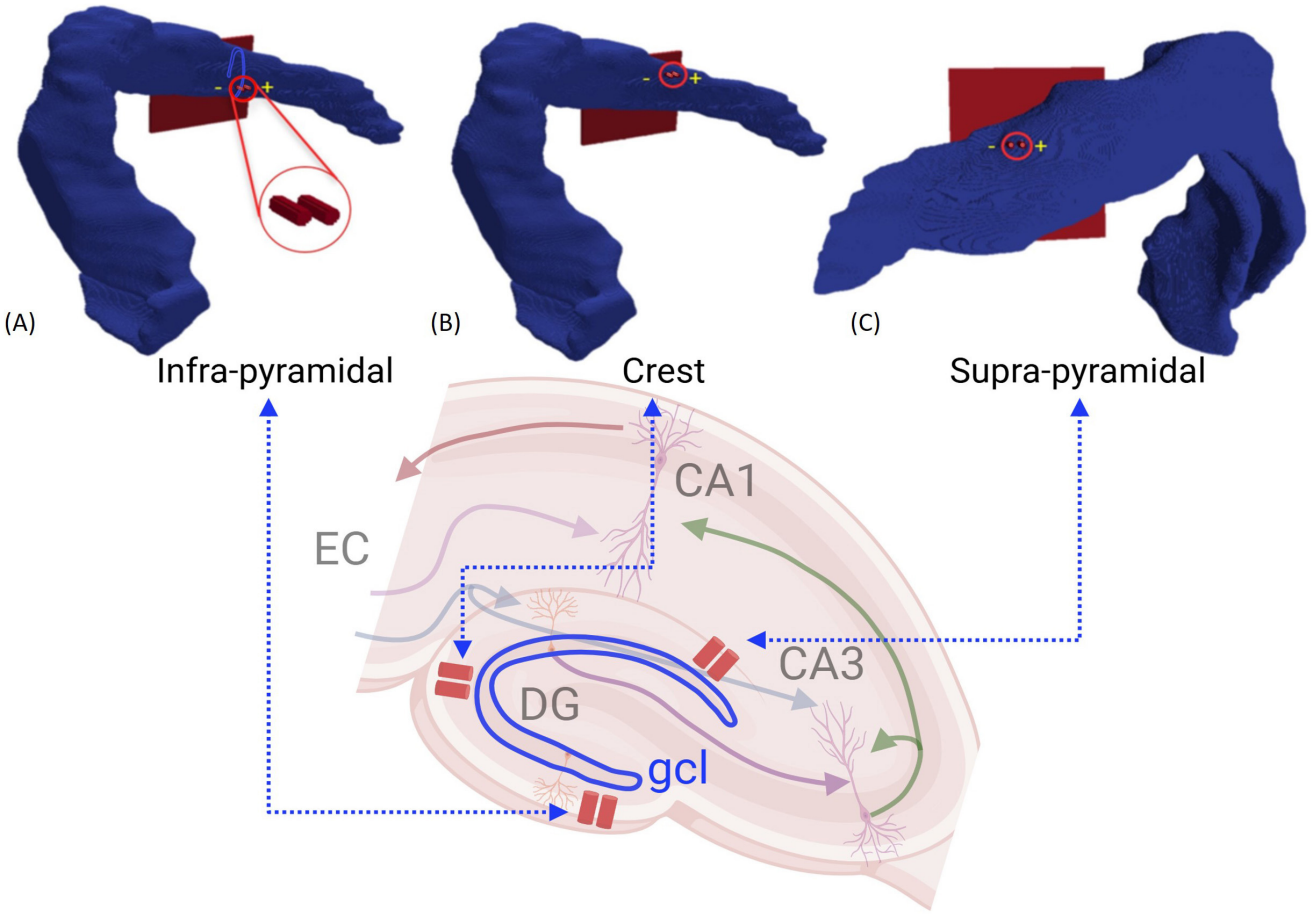
Stimulation locations in the 3D model: **(A)** Infrapyramidal, **(B)** Crest, and **(C)** Suprapyramidal. A biphasic, charge-balanced, and square-wave impulse of 1 ms width was applied to evaluate the activation of EC axons that consequently leads to synaptic activation of GC.

**Figure 5:**
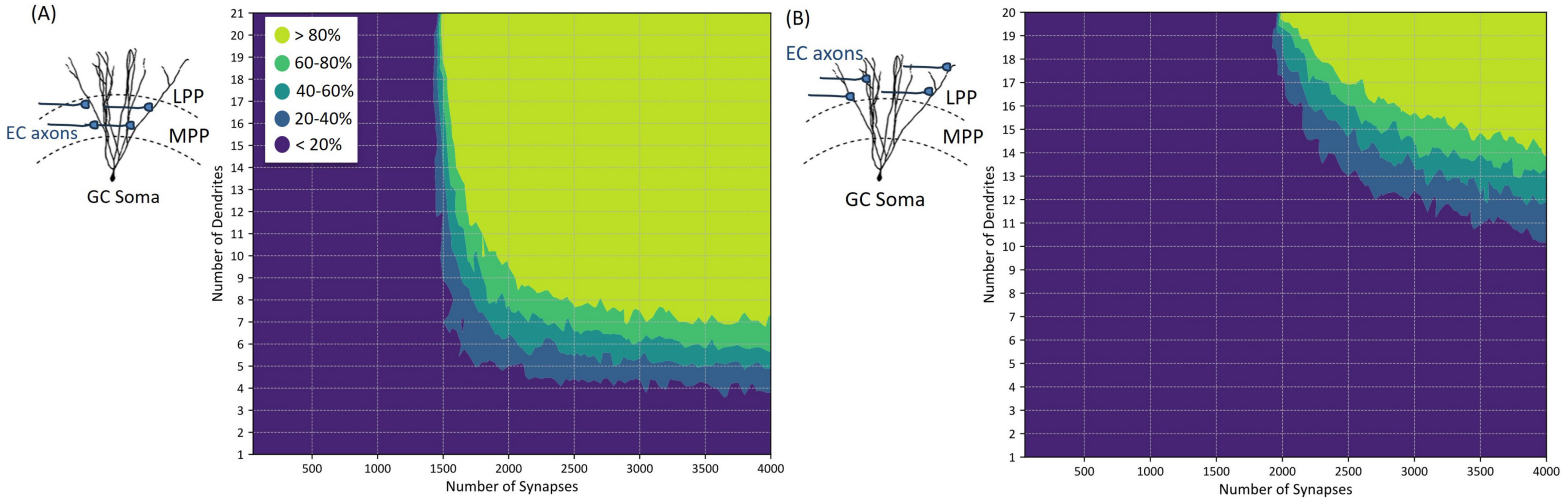
Characterization of dendritic integration of synaptic inputs in granule cells (GCs) and corresponding GC activation threshold. The plots depict the relationship between the number of active synaptic inputs and the probability of generating an action potential. Notably, the number of dendrites on which these active synapses are distributed influences the activation threshold, with more distributed configurations (i.e., increased number of dendrites) resulting in a slight decrease in the number of active synapses needed to generate an action potential. **(A)** Illustrates the synaptic connections of EC axons to GC at the medial perforant path (MPP), while **(B)** illustrates the situation where active synaptic connections are located at the lateral perforant path (LPP).

**Figure 6:**
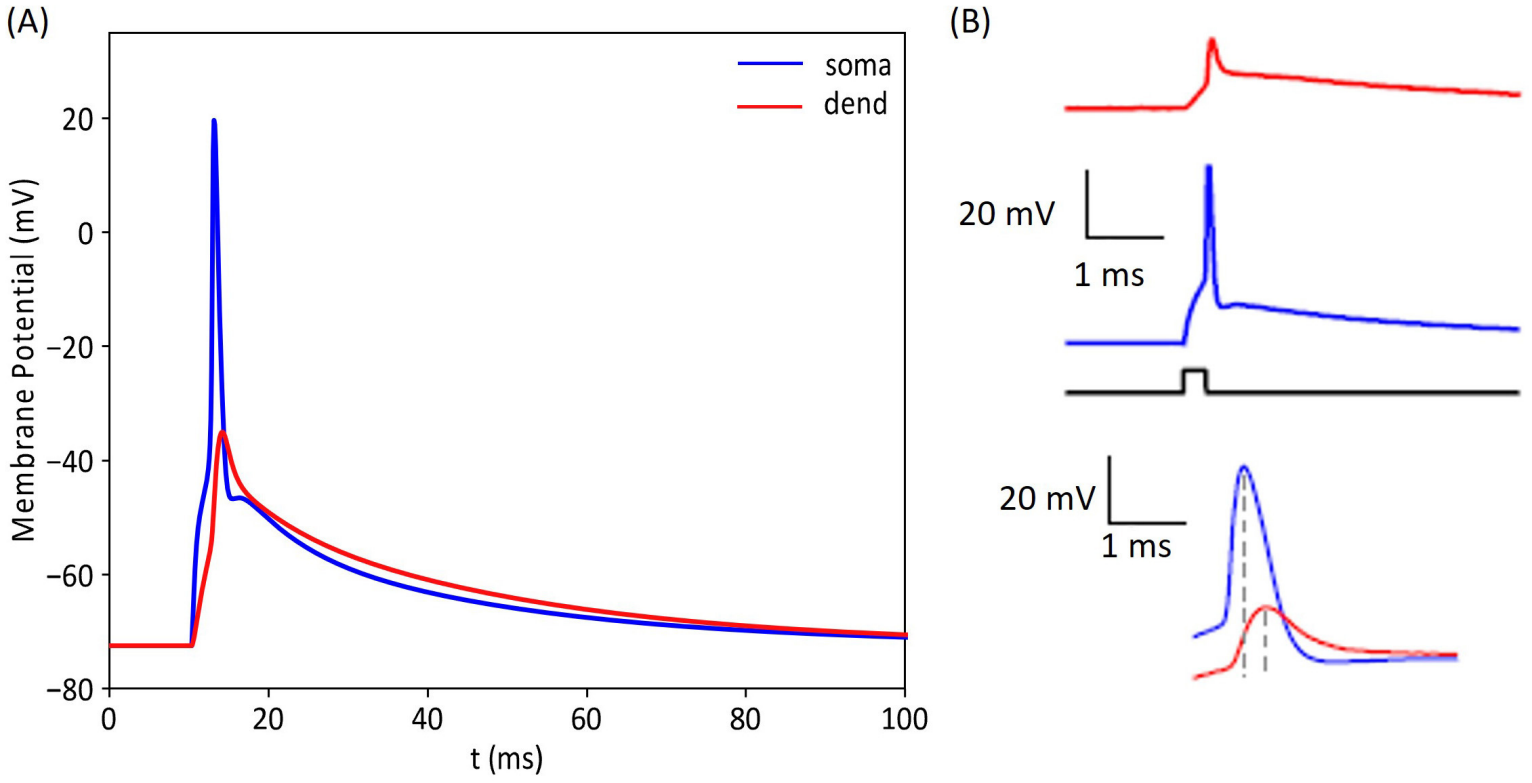
Comparison of intracellular voltages recorded experimentally and with our computational model. **(A)** illustrates the membrane potential response of our GC model at the soma and in a proximal dendrite. For this result, we used 1,800 synaptic inputs distributed on 20 dendrites. **(B)** shows the corresponding experimental activation profiles reported by Krueppel et al. (2011). The intracellular voltage traces obtained with the model are in accordance with experimental recordings, indicating that our simulated GC model replicates the response of its biological counterpart, highlighting the model's ability to accurately predict the cell's behavior.

**Figure 7:**
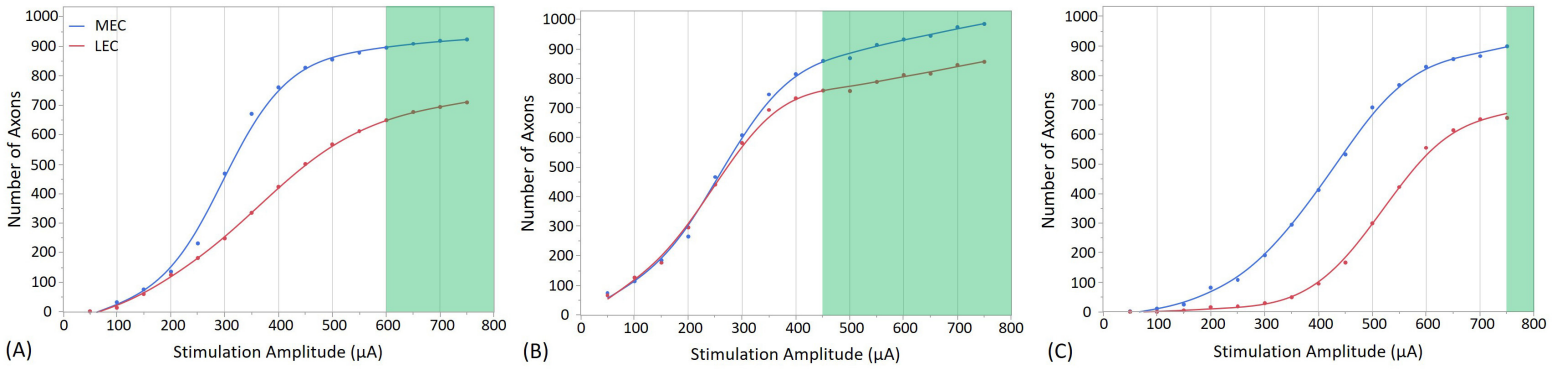
Stimulation effects with the electrode placed in the cell body layer of the dentate gyrus at different positions. **(A)** shows the stimulation at the Crest, **(B)** at the Infra-pyramidal, and **(C)** at the Supra-pyramidal region. The stimulation amplitude ranges from 50 to 750 μA. The green area on the plot represents the amplitudes at which the EC synaptic connections with GCs reach a threshold, resulting in the firing of the GCs. In addition to highlight the effect of stimulation amplitude, this figure illustrates how electrode placement within these specific regions influences the activation of neural tissue in the dentate gyrus.

**Figure 8:**
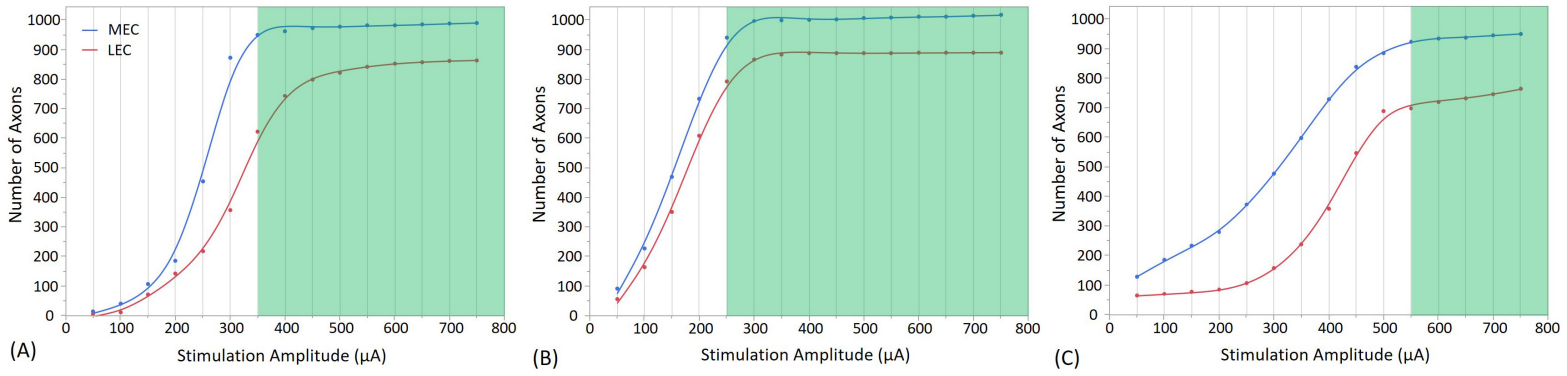
Direct and indirect (i.e., synaptic) consequences of electrical stimulation when the electrode is placed in the Molecular Layer of the Dentate Gyrus at three different positions, with different stimulation amplitudes. **(A)** shows the stimulation at the Crest, **(B)** at the Infra-pyramidal, and **(C)** at the Supra-pyramidal region. The stimulation amplitude ranges from 50 to 750 μA. The Y axis represents the number of axons activated. The green area on the plot represents the amplitudes at which the EC synaptic connections with GC reach a threshold, resulting in the firing of the GC.

After constructing the models for various stimulation sites as depicted in Figure 4, simulations were performed to assess the effect of multiple parameters on the granule cells response. This process involved several steps. First, we examined the effect of synaptic integration on GC activation, focusing on how synaptic inputs integrate along the dendritic tree to shed some light on how different synaptic activation profiles shape the overall cellular response (Figure 5). Next, we validated the GC synaptic activation model by comparing the simulation results with experimental data, ensuring that the model reproduces the granule cell behavior as observed experimentally when they are synaptically stimulated (Figure 6). Finally, we simulated the application of an electric field using the AM-NEURON model to analyze the resulting axonal activation and the subsequent sensitivity of GC activation for different stimulation locations and pulse amplitudes (Figures 7, 8). This final step aimed to identify parameters that result in the most efficient granule cell activation with minimal power consumption, thereby yielding useful predictions to guide stimulation device development and placement for practical applications. Further details for each of these steps are provided in the following sections.

### 3.1 Granule cell synaptic integration and model validation

To characterize granule cells (GCs) activation in response to synaptic inputs, we first evenly place synapses on the number of dendritic branches to generate an action potential. We examine the effect of synaptic clustering on the GC dendrites, as well as the consequences resulting from their location on the dendritic tree. To do so, we subdivide the dendritic tree into two regions, the middle third in which synapses are formed with axons originating from the medial perforant path (MPP), and the outer third region in which synapses are formed with axons originating from the lateral perforant path (LPP), illustrated in Figure 1.

The granule cell model consists of 21 dendritic segments on the medial side and 20 dendritic segments on the lateral side. Figure 5A illustrates the synaptic integration within the medial perforant path (MPP) segments of GC, while Figure 5B shows the synaptic integration for the lateral perforant path (LPP) segments.

To assess the probability of granule cell action potential generation, we conducted 100 simulations, each with variable numbers of synapses activated evenly across different dendritic segments at both MPP and LPP positions. In each simulation, the number and location of active synapses were randomized to capture variability in synaptic input patterns and the stochastic nature of synaptic transmission. This probabilistic approach allows us to quantify action potential generation likelihood across diverse synaptic activation scenarios, accounting for inherent randomness in synaptic responses and dendritic integration.

Figure 5 illustrates that the location and clustering properties of active synapses affect the probability of activating the GC, with a threshold that varies based on the number of synapses and dendritic connections. Specifically, Figure 5A indicates that at least 1,500 active synapses distributed on all 21 medial dendritic segments are needed to activate the GC with a probability <80%. When the number of medial synapses activated exceeds 2,000, a more clustered distribution is sufficient to result in a similar activation (activated synapses should be distributed on more than 9 dendritic segments).

Similarly, Figure 5B demonstrates that more than 2,000 synchronous synaptic inputs distributed on at least 20 lateral dendritic segments of the GC are required to activate the GC with a probability <80%. Additionally, as the number of active LPP synapses increases, less segments are required for AP generation. For example, with over 2,500 active synapses, 17 dendritic segments (or more) are sufficient to elicit the same probability of activation.

To validate the response of our GC model to synaptic inputs, we recorded the intracellular voltage at multiple locations within the cell and compared these recordings with experimental results. Figure 6A illustrates this validation process, showing the simulation responses obtained at the soma (blue) and one of the dendrites (red). The simulated voltages align with experimental findings from a previous study conducted by Krueppel et al. (2011) obtained in response to similar synaptic stimulation.

### 3.2 Granule cell activation threshold across different stimulation sites

A primary objective of this modeling effort was to identify electrode placements and stimulation strategies to elicit a specific response from hippocampal tissue. This study details the results of multiple excitation sites, with the aim of determining the effect of electrode position and stimulation amplitude on GC activation.

In this manuscript, two electrode placement setups separated by 200 μm were evaluated. The first setup involved positioning the electrode within the granule cell layer. This region is crucial because it houses the densely packed cell bodies of GCs, which are the principal neurons of the dentate gyrus. In the second setup, electrodes are placed in the molecular layer, where significant synaptic connections are formed between the medial and lateral EC axons and the dendrites of the granule cells. Furthermore, three primary stimulation sites were considered for the analysis: the crest, infra-pyramidal, and supra-pyramidal regions, shown in Figure 4. For each of the three electrode sites, we systematically evaluated multiple electrode positions to identify the optimal placement. Specifically, we measured the distance between the electrode tip and all axons connected to the target granule cell, selecting positions that minimized the distance from the axon segments and measuring the mean segment diameter at these positions. From all evaluated positions, we selected the electrode placements where the diameter closely matched among all three stimulation sites. This ensures a consistent comparison across locations and maximizes proximity to first branching point. This approach was chosen because the first bifurcation point has the largest axon diameter, which enhances its responsiveness to electrical stimulation as larger axons require less current to reach the activation threshold compared to smaller ones, making them more responsive to electrical stimuli (Rattay, 1999; McIntyre et al., 2002; Verveen, 1962). The results indicated that, for electrodes placed in the cell body layer, the mean distances from all axon segments to a single granule cell were 70.86 μm at the crest, 66.84 μm at the infra-pyramidal, and 105.96 μm at the supra-pyramidal positions, with the corresponding mean axon segment diameters being 1.03 μm, 0.99 μm, and 1.08 μm, respectively. For electrodes placed in the molecular layer, the mean distances were 14.35 μm at the crest, 23.33 μm at the infra-pyramidal, and 40.86 μm at the supra-pyramidal positions, with mean axon segment diameters of 1.1 μm, 1.04 μm, and 1.02 μm, respectively.

Figures 7, 8 present the outcomes of stimulating the GC layer when electrodes are positioned in the GC cell body layer and the molecular layer, respectively. The GC cell body layer primarily contains neuron cell bodies responsible for receiving and processing inputs, while the molecular layer is rich in dendritic and axonal processes that facilitate complex synaptic interactions. These figures detail the activation patterns of MEC and LEC axons, which subsequently lead to GC response via synaptic activation at various stimulation sites and at different stimulation amplitudes.

Stimulation with an amplitude of 600 μA at the crest electrode site activated 88% of MEC axons and 73% of LEC axons (from the total number of 1,909 EC axons that project to one single GC), as shown in Figure 7A. Additionally, granule cell activation occurs at the infra-pyramidal location with a stimulation amplitude of 450 μA, where 85% of MEC axons and 86% of LEC axons were activated, as depicted in Figure 7B. Furthermore, granule cell activation is also observed at the supra-pyramidal location with a stimulation amplitude of 750 μA, resulting in the activation of 89% of MEC axons and 74% of LEC axons, as shown in Figure 7C.

In Figure 8A, at a stimulation amplitude of 350 μA at the crest electrode site, 93% of MEC axons and 70% of LEC axons were activated. Granule cell activation also occurs at the infra-pyramidal location with a stimulation amplitude of 250 μA, where 92% of MEC axons and 88% of LEC axons were activated, as depicted in Figure 8B. Additionally, granule cell activation is observed at the supra-pyramidal location with a stimulation amplitude of 550 μA, resulting in the activation of 90% of MEC axons and 76% of LEC axons, as shown in Figure 8C.

In all six positions, the MEC count exceeds the LEC count, as the electrode tip is positioned closer to the middle third of the granule cell dendritic tree, facilitating greater MEC activation.

These results highlight the significant impact of varying stimulation amplitude and electrode placement on the number of activated MEC and LEC axons. They also suggest that among these 3 electrode sites, stimulation at infra-pyramidal locations yields the lowest stimulation amplitude threshold. This is true when the electrode is placed both in the cell body layer and the molecular layer. Higher stimulation amplitudes are required for stimulation in the crest, and the highest stimulation amplitudes are needed in the supra-pyramidal blade.

## 4 Discussion

The work presented here describes a detailed 3D model of the Dentate Gyrus, featuring realistic, myelinated, three-dimensional perforant path axons and the direct and indirect consequence of electrically stimulating these axons on the downstream activation of DG granule cells. This development extends prior research by Bingham et al. (2018), by enhancing axonal morphologies through the inclusion of axon diameter tapering and incorporating realistic biophysical properties of myelinated axons. Earlier studies have either focused on bulk-tissue-level modeling without considering network dynamics or examined smaller networks using multi-compartmental neuron models. In contrast, this study employs a hybrid, multi-scale (from tissue to network to cell to subcellular scales) AM-NEURON model, encompassing highly detailed axonal morphologies and biophysical properties, and the resulting electrical activity of DG granule cells. While finite element methods (FEM) often require significant computational resources—especially for complex or large-scale problems—resulting in long simulation times and substantial memory demands, the AM-NEURON platform we have developed is inherently capable of modeling both complex, large-scale heterogeneous biological tissues and micro-scale, biophysically, and morphologically accurate neuron models. Its multiphysics capabilities help guide the design of electrical stimulation strategies to enhance the effectiveness of current hippocampal prosthetic systems, ultimately steering the development of the next generations of prostheses. By analyzing the sensitivity of these models to variations in dielectric properties and electrical stimuli, we can reveal essential properties of the hippocampal system. These insights are crucial for guiding the design of more efficacious therapeutic interventions and devices.

### 4.1 Enhancements of the model relative to prior research

Previous investigations utilizing the NEURON model that underpins this study have identified the spontaneous formation of spatiotemporal clusters of activity within the DG driven by synaptic inputs (Hendrickson et al., 2015). The present model incorporates a detailed three-dimensional representation of the dentate gyrus region, integrating realistic myelinated axons for EC projections. This enhancement enables the characterization of the effect of exogenous electrical stimulation as an additional input that stimulates the hippocampal network. Consequently, the model enables the investigation of stimulation thresholds and the spatiotemporal patterns of activity generated in response to electrical stimulation, providing an accurate visualization of the active signal propagation across the transverse structure.

Key structural features such as diameter, tapering, branching points, and geometric orientation relative to the applied field can significantly influence the local polarization of membrane compartments. For example, experimental studies have shown that granule cell mossy fiber axons exhibit substantial tapering along their length, with proximal diameters around 1 μm gradually narrowing to ~0.5 μm in distal segments (Schmidt-Hieber et al., 2008). This gradual tapering affects axial resistance and local current flow, which in turn modulates the sensitivity of axonal compartments to electric fields.

Real axons often display non-linear tapering profiles, diameter irregularities, and varicosities—features that can produce local hotspots of depolarization or alter conduction properties (Wybo et al., 2021). Additionally, axonal bifurcations introduce impedance mismatches that can change spike initiation and propagation thresholds, particularly in the presence of spatially nonuniform extracellular fields (McIntyre et al., 2002; Rattay, 1999). In this model, we implemented a simplified linear tapering profile aimed at replicating the general trend observed experimentally while capturing first-order effects of morphology on excitability.

The integration of 3D EC axons with realistic myelination and tapering (Bingham et al., 2020; Aberra et al., 2018; Hu et al., 2009), following accurate anatomical topography, enhances the model's ability to predict spatiotemporal responses to extracellular stimulation. Investigations of the effect of electrical stimulation often focus on the immediate and local responses of directly stimulated neurons. For example, they might determine that an action potential begins at a specific axonal segment near an electrode. However, these studies typically do not account for how this activation propagates through the neural network or affects other connected neurons. In contrast, our hybrid, multi-scale AM-NEURON model reveals broader activation patterns by simulating both the local initiation of action potentials and their subsequent propagation throughout complex neural circuits by taking into account the statistical properties of the extent of the axonal arbor as established by Tamamaki and Nojyo (1993). These patterns emerge from the initial stimulation characteristics, the complex axonal propagation, the resulting synaptic events and their integration along the granule cells dendritic tree.

### 4.2 Computational requirements and factors

When selecting the modeling method described in this study, addressing the computational load is a significant challenge. Simulating extensive networks of intricate neuronal models demands substantial computational power. For this work, the model was parallelized and simulated on a high-performance computing cluster with 4,040 processors available to the authors. The time needed to generate 3D axonal morphologies, as depicted in Figures 3C, D, varies significantly depending on branching features of generated topologies and the number of connections with the resulting GCs. This process can range from 8 h to several days on CARC, depending on the complexity and number of synaptic connections formed. The voltage matrix produced by the AM model, illustrated in Figure 4, generates an extensive dataset exceeding 6 GB for each configuration. Simulations for the results presented in Figures 7, 8 generated over 450 GB of data to analyze the response of each granule cell across all configurations and stimulation amplitudes. The simulations required ~100 h of processor time to simulate just under 2 s of network activity using 1,909 processors.

The increasing complexity of parametric models, despite their interpretability, creates substantial computational challenges. As future models incorporate more detailed topology, longer simulated times, intricate stimulation protocols, and feedback potentials, they will likely demand resources beyond current capacities that will require further optimization. To alleviate computational burden, parallel processing of axons using multiple-input/multiple-output modeling has been implemented to evaluate the synaptic activation at the GC. There is a pressing need for algorithms that simplify the complexity of axonal branching (while retaining sufficient realism and predictive power); the reimplementation/simulation of these models on GPU technology may also help reduce the computational burden.

### 4.3 Energy-efficient activation of entorhinal cortex axons

To determine the number of EC axons needed to activate a GC, it is essential to identify the number of synaptic connections necessary to reach the activation threshold. Figure 5 indicates that at least 1,500 synaptic connections from medial entorhinal cortex (MEC) axons; similarly 2,000 synapses from the lateral entorhinal cortex (LEC) axons are required. Figure 6 demonstrates the validity of the granule cell (GC) model, particularly its ability to integrate synaptic inputs and trigger action potentials in both dendritic and somatic regions. These simulation results are in close agreement with experimental findings from Krueppel et al. (2011), supporting the model's accuracy in replicating the physiological mechanisms driving action potential initiation across these regions.

In Figures 7, 8, an extracellular stimulation paradigm was applied to determine the minimum number of EC axons needed to activate a GC. The results reveal that ~1,550 combined MEC and LEC axons are required for GC activation at the crest stimulation site (Figures 7A, 8A). However, when stimulating the infra-pyramidal position at a lower activation threshold, around 1,630 to 1,720 MEC and LEC axons are activated (Figures 7B, 8B). The findings also indicate that placing the stimulation electrodes closer to the molecular layer, rather than the cell body layer, significantly reduces the activation threshold. This reduction is likely due to the lower electrical resistivity of the molecular layer and closer proximity to axons.

Unsurprisingly, increasing stimulation amplitude results in more MEC and LEC axons being activated, leading to a greater number of synaptic inputs to the GC. Notably, GC activation thresholds in the six positions required activation of at least 75% of all axons. As stimulation amplitudes are increased above threshold, the number of activated axons increases, and eventually plateaus as it reaches the total number of excitable axons (1,909 axons in total, with 1,016 MEC and 893 LEC axons). Once past the threshold, the number of activated axons projecting to the target GC increases only marginally (particularly for the Molecular Layer positions as outlined in Figure 8). Although increasing the amplitude further recruits only a small number of additional axons projecting to the same GC, it may activate a larger number of axons connected to other GCs. This particular aspect was not investigated in this study. Of importance, higher stimulation amplitude could increase the risk of tissue damage and potentially reduce overall stimulation efficacy. In addition, an important factor driving differences in activation thresholds across all stimulation sites appears to be the variability in activation rate of LEC axons compared to MEC axons. For example, at the Crest location (Figures 7A, 8A), both configurations show that LEC axon activation reaches only about 620 axons at threshold (70% of the total number of LEC axons), while about 90% of MEC axons are activated. The LEC axons exhibit additional activation potential beyond the activation threshold, indicating a capacity for further recruitment. In contrast, a larger portion of MEC axons are activated at the threshold. This difference is also noticeable in the infra-pyramidal position, especially when the electrode is situated in the molecular layer (Figure 8B). At the supra-pyramidal position, the number of activated LEC axons remains comparable for both electrode placements (Figures 7C, 8C). Finally, axonal recruitment rates are higher when the electrode is positioned in the molecular layer, suggesting that electrode placement in this layer results in higher overall stimulation efficiency.

When combining observations from Figures 7, 8, the lowest stimulation amplitude required for activation is achieved when the electrode is positioned in the molecular layer of the infra-pyramidal region at 250 μA, followed by the crest position at 350 μA, and finally the supra-pyramidal position at 550 μA. The difference between electrode placements in the cell body layer and the molecular layer was most pronounced at the supra-pyramidal position. Of note, the stimulation amplitudes in this study are occasionally higher than those typically used in clinical settings, especially for activation of large and myelinated fibers. The activation thresholds could be significantly higher for small unmyelinated axons, which are theoretically less excitable (Nowak and Bullier, 1998). This study indicates that large evoked potentials can be elicited with 1 ms pulses at 250 μA. However, not all stimulation locations in this study exhibited such low excitation thresholds, and the response curves only began to plateau at much higher-amplitude pulses, such as those in the cell body layer.

### 4.4 Future works and limitations

Notably, this study makes use of a limited network of over 8,000 entorhinal cortex (EC) axons that project to 11 granule cells, representing a small subset of the projections from EC to DG and DG granule cells. Subsequent studies will expand the investigation to encompass the entire dentate gyrus (that is, more than 100,000 EC axons and more than 1.2 million granule cells), and cells in the cornu Ammonis (CA) region, specifically CA1 and CA3. Such model will enable to characterize the effect of electrical stimulation on the spatiotemporal patterns of activity as they propagate in the entire hippocampal trisynaptic pathway.

Notably, it would be advantageous to extend the methodology and incorporate local field potentials, ephaptic coupling, and other activity-dependent extracellular dynamics across different hippocampal regions (Elbohouty et al., 2013; Fritz and Gardner-Medwin, 1976; Shifman and Lewis, 2019). Adding these elements would provide a more comprehensive view of the hippocampal system, its intricate neural interactions and its response to electrical stimulation. Importantly, this expanded model would also benefit from easier validation with respect to experimental observations (e.g., local field potentials).

This model includes only AMPA-mediated excitatory inputs to focus on the core dynamics of excitatory propagation following a single stimulation event while minimizing computational complexity. NMDA receptors and inhibitory inputs, such as those from Molecular Layer Perforant Pathway (MOPP) cells, were excluded due to their relatively limited contribution under single-pulse conditions and their more prominent role in modulating responses to repeated or patterned inputs (Li et al., 2013; Ewell and Jones, 2010). Future work may incorporate these mechanisms to provide a more complete representation of synaptic and network integration, particularly in the context of multiple spatiotemporal stimulation patterns.

Of importance, critical neural components of the dentate gyrus were not included in the model presented here, particularly interneurons. DG contains several types of GABAergic interneurons, including somatostatin-positive interneurons (SOMIs) and hilar-perforant-path-associated interneurons (HIPP cells). These interneurons provide different forms of inhibition to granule cells: feedback inhibition by receiving input from granule cells and then inhibiting them, or feedforward inhibition by receiving input from the entorhinal cortex and inhibiting granule cells before they can activate, thereby modulating population dynamics. Integration of interneurons into the model should be prioritized as research focus shifts toward investigating the propagation of spatiotemporal patterns of activity in the hippocampal network.

## 5 Conclusion

This study presents a multiscale computational model that integrates the admittance method (AM) used to compute the electric field generated by a stimulating electrode in conjunction with realistic 3D reconstructions of EC axons and the DG granule cells onto which they project. The results obtained with this model highlight the effect of electrode positioning and stimulation amplitude on neural activation. The results indicate that optimizing these parameters can greatly affect stimulation efficiency and the resulting direct and indirect neural activation. The computational approach presented constitutes a robust framework to help guide the design of devices such as multi-electrode arrays to more effectively interface with the brain, thereby presenting an innovative framework for testing prototypes of prostheses aimed at interfacing with neural tissue.

## Data Availability

The raw data supporting the conclusions of this article will be made available by the authors, without undue reservation. A pointer to its location can be found at: https://itemsusc.org/resources/.
